# The First Bilateral Staged Oxford Cementless Unicompartmental Knee Arthroplasty in Louisiana Following FDA Approval: A Case Report

**DOI:** 10.7759/cureus.99273

**Published:** 2025-12-15

**Authors:** Nicholas M Villar, Grace Lee, Joseph Vo, Cameron Cluney, Lee A Dennis, Jenna Dittmar

**Affiliations:** 1 Orthopedics, Edward Via College of Osteopathic Medicine, Monroe, USA; 2 Ophthalmology, Edward Via College of Osteopathic Medicine, Monroe, USA; 3 Emergency Medicine, Edward Via College of Osteopathic Medicine, Monroe, USA; 4 Medicine, Edward Via College of Osteopathic Medicine, Monroe, USA; 5 Anatomy, Edward Via College of Osteopathic Medicine, Monroe, USA

**Keywords:** medial compartment osteoarthritis of knee, orthopedic implants, orthopedic surgery, osteoarthritis, oxford unicompartmental knee arthroplasty

## Abstract

We present the case of a 63-year-old woman with bilateral medial compartment osteoarthritis who underwent the first staged bilateral cementless Oxford unicompartmental knee arthroplasty (OUKA) performed in the state of Louisiana shortly after its FDA approval in 2024. The patient had a five-year history of progressively worsening knee pain refractory to conservative therapy, including corticosteroid injections and physical therapy. Imaging confirmed isolated medial compartment disease with preserved lateral compartments and intact cruciate ligaments. Following the right OUKA, she demonstrated rapid recovery, full extension, and near-complete resolution of pain within four weeks. Due to persistent pain in the contralateral knee, a left OUKA was performed six weeks later with similarly favorable results. Postoperative imaging confirmed appropriate prosthesis positioning bilaterally without evidence of loosening, migration, or malalignment. The patient resumed full ambulation and reported restoration of functional capacity. This case highlights the early postoperative benefits and potential long-term promise of cementless OUKA, including faster recovery, reduced surgical time, and avoidance of cement-related complications. The implant’s titanium and hydroxyapatite coating may further enhance biological fixation and durability. As cementless technology gains traction in the United States, continued follow-up and longitudinal studies will be essential to confirm its long-term survivorship and clinical efficacy compared to traditional cemented designs.

## Introduction

Unicompartmental knee arthroplasty (UKA) is an alternative surgical technique used to treat osteoarthritis affecting a single compartment of the knee, most commonly the medial compartment [1]. In contrast to total knee arthroplasty (TKA), which treats the lateral, medial, and patellofemoral compartments of the knee, UKA preserves native bone and ligaments, offering a less invasive approach [2]. UKA was intended for patients with isolated anteromedial compartment osteoarthritis or avascular necrosis with ample bone stock and intact cruciate ligaments and medial collateral ligament [1]. Although concerns previously existed regarding younger patients with high activity levels or higher body mass index (BMI), these factors are no longer considered contraindications due to improvements in implant design and expansion of the traditional indications for UKA [1]. Since its introduction in the 1970s, UKAs have been heavily debated for their effectiveness and durability because of high failure rates related to aseptic loosening, persistent pain, and subsequent revision surgeries and conversion to TKA [1]. However, modifications and improvements in implant design and surgical technique have renewed interest in UKA as an effective treatment option. Recent innovations, including cementless fixation, have further contributed to its resurgence through improved implant fixation, decreased risk of loosening, an enhanced minimally invasive approach, and faster recovery [3].

The Oxford unicompartmental knee arthroplasty (OUKA) system was introduced in 2004 and has been one of the most widely studied and utilized designs worldwide [3]. After 20 years of research and over 300,000 procedures performed in Canada, Europe, the Middle East, Africa, and Asia, Zimmer Biomet’s cementless version received FDA approval in November 2024 [4,5]. Studies have shown that cementless OUKA is associated with decreased long-term revision rates, as well as improved fixation and postoperative functional outcomes, compared with its cemented counterpart [6,7]. Despite these promising results, continued evaluation of clinical outcomes remains essential to better define the durability associated with this new and evolving technique.

We present a patient who underwent bilateral OUKA shortly after FDA approval, making her the first in the state of Louisiana to undergo this procedure twice within a three-month span. This case is notable for the patient’s rapid postoperative recovery, early return to function, and high overall satisfaction, outcomes that highlight the potential clinical benefits of cementless OUKA in a market still dominated by TKA. 

## Case presentation

A 63-year-old female with a past medical history of osteoarthritis of the right hip, migraine headaches, and hypothyroidism presented with bilateral knee pain, right greater than left, of five years’ duration with progressive worsening. The pain was localized to the medial aspect of both knees and significantly limited her activities of daily living and overall quality of life. She noted that she was no longer able to enjoy her regular activities or remain active without significant pain, causing her to stop. Past surgical history included total hip arthroplasty of the right hip and tubal ligation. Her current medications included celecoxib (Celebrex) 200 mg for knee pain, eletriptan hydrobromide (Relpax) 40 mg for migraines, and levothyroxine sodium (Synthroid) 112 mcg for hypothyroidism. Regarding her knee pain, she had previously undergone multiple corticosteroid injections and a course of physical therapy consisting of two sessions per week for six weeks to improve strength and range of motion, which she noted provided only minimal relief. The patient denied any history of drug or tobacco use. Having exhausted conservative measures, she sought surgical options for definitive treatment.

Physical examination demonstrated that both knees corrected to normal alignment with valgus stress, were minimally tender to palpation, and had negative anterior and posterior drawer tests. The patient also had a normal gait and denied pain with hip range of motion in either leg, ruling out the possibility of referred pain from prior hip surgery.

**Figure 1:**
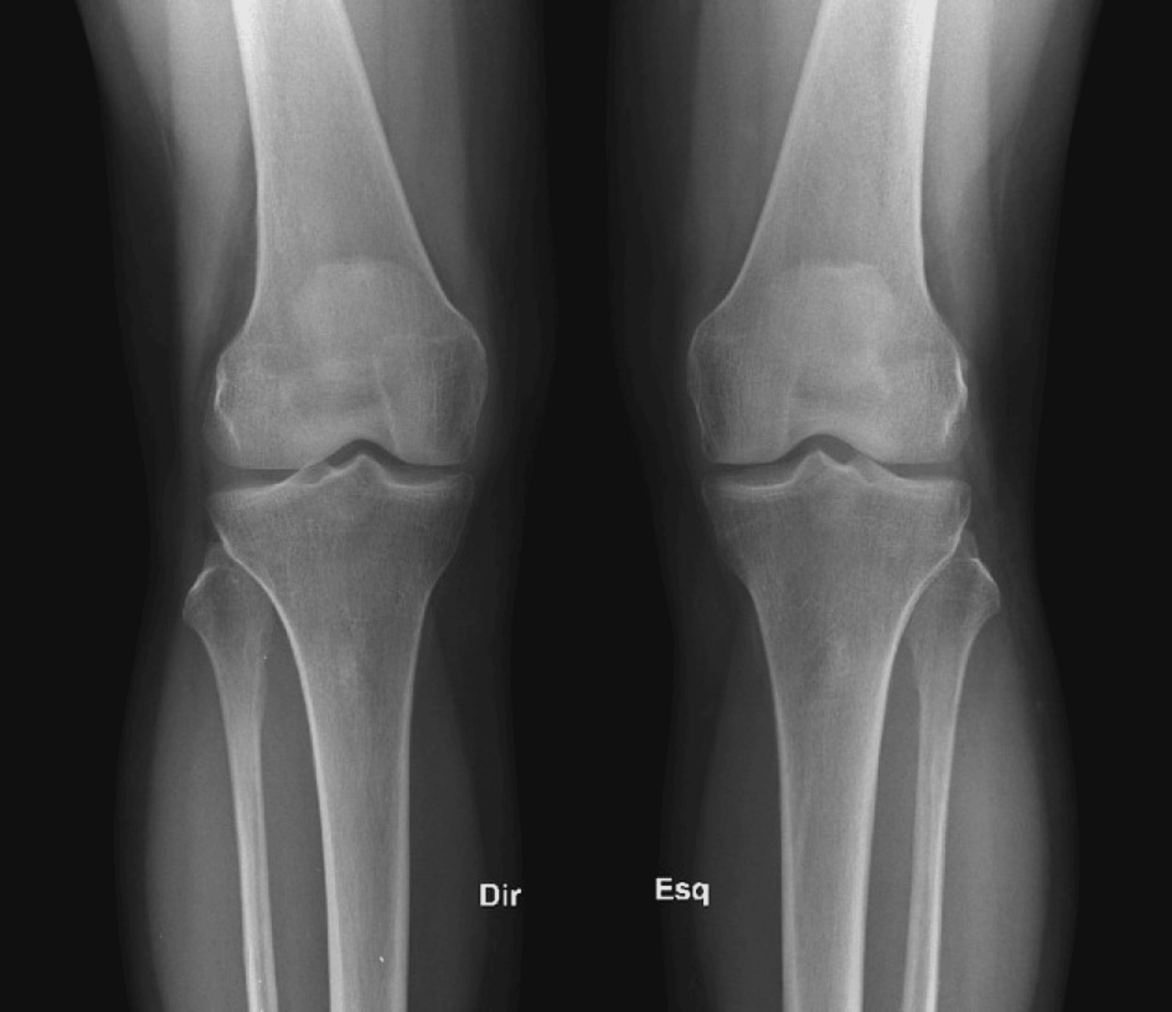
Anteroposterior view of both knees showing medial compartment narrowing consistent with end-stage unicompartmental osteoarthritis.

**Figure 2:**
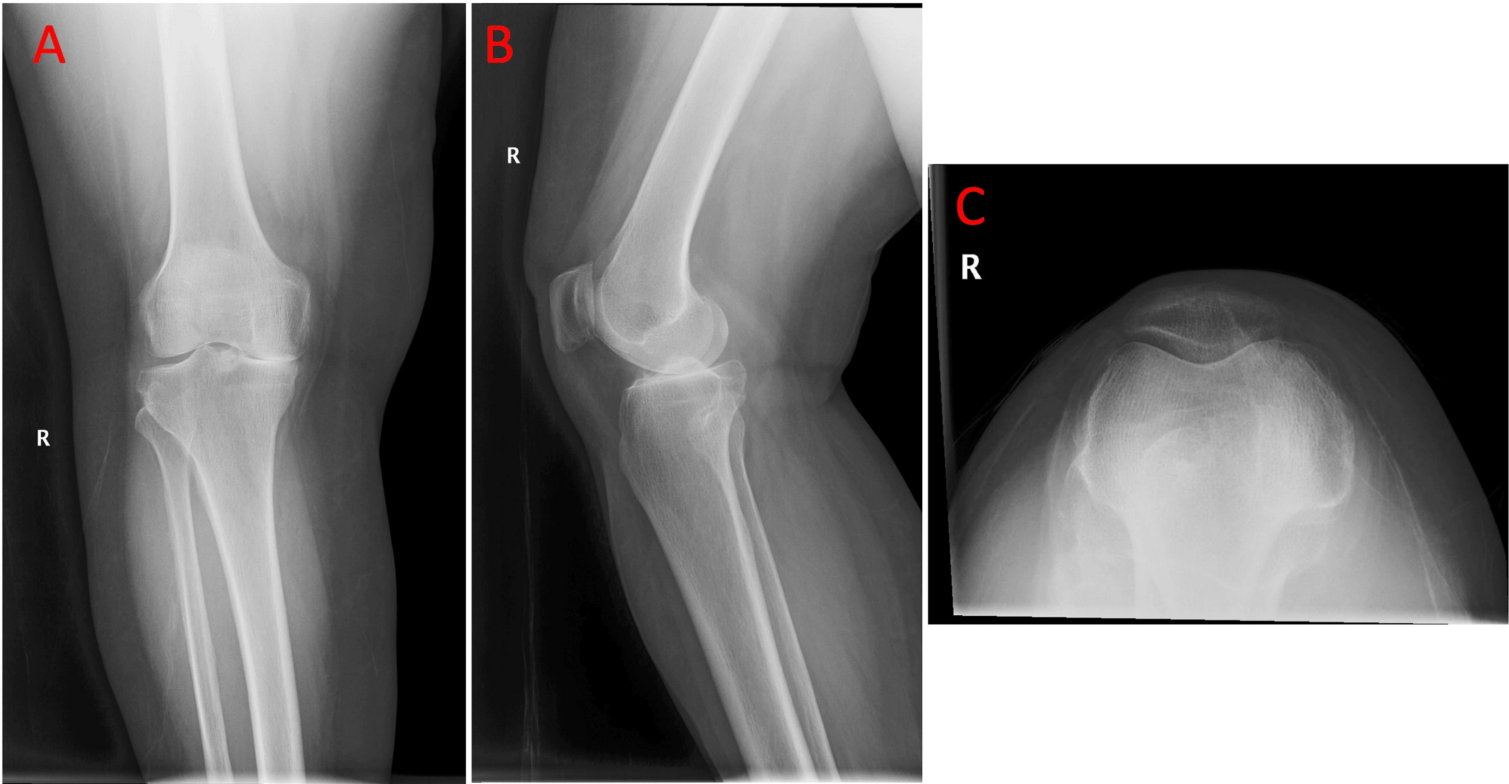
a) Anteroposterior view of the right knee showing medial compartment narrowing consistent with end-stage unicompartmental osteoarthritis. b) Lateral view of the right knee showing overlapping femoral condyles and preserved posterior tibial slope, providing evidence of an intact anterior cruciate ligament. c) Sunrise view of the right knee showing preserved patellofemoral joint space without bone loss.

**Figure 3:**
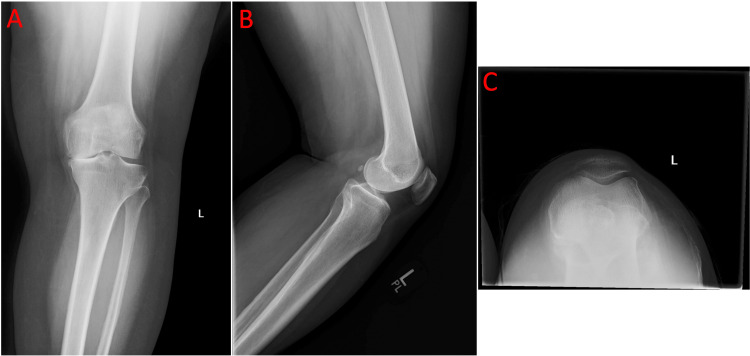
a) Anteroposterior view of the left knee showing medial compartment narrowing consistent with end-stage unicompartmental osteoarthritis. b) Lateral view of the left knee demonstrating overlapping femoral condyles and preserved posterior tibial contour, providing evidence of an intact anterior cruciate ligament. c) Sunrise view of the left knee showing medial facet narrowing without bone loss.

Four-view radiographs of both knees demonstrated bone-on-bone osteoarthritis of the medial compartments, preservation of the lateral compartment joint spaces, and no anterior tibial tilt on the lateral view, suggesting intact anterior cruciate ligaments (Figure 1, Figure 2, and Figure 3).

Surgical options, including OUKA and TKA, were discussed in detail. Because imaging demonstrated medial compartment osteoarthritis with no anterior tibial tilt, suggesting sufficient ligamentous stability, both knees were appropriate candidates for OUKA. After discussion of surgical options, the patient elected to undergo surgery on the right knee first, as it caused the most pain, and preferred OUKA due to its less invasive approach and faster recovery [3]. She was subsequently scheduled for surgery after obtaining medical and cardiac clearance. Postoperatively, she was prescribed aspirin 81 mg to reduce the risk of blood clot formation and hydrocodone-acetaminophen (Norco) 7.7-325 mg for pain control.

**Figure 4:**
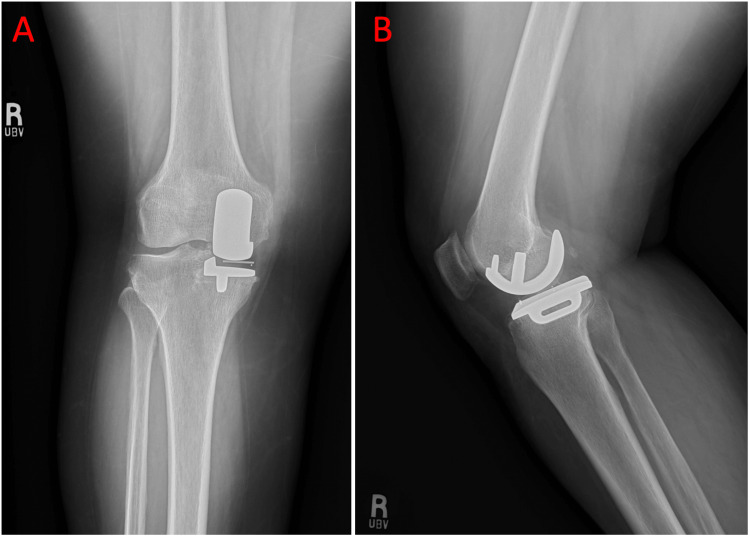
a) Postoperative anteroposterior view of the right knee showing an intact prosthesis without evidence of loosening or migration. b) Postoperative lateral view of the right knee showing an intact prosthesis without evidence of loosening or migration.

At her two-week postoperative follow-up, she reported mild neuropathic pain radiating from the knee to the hip but was ambulating independently. Physical examination demonstrated knee flexion beyond 95° and full extension without discomfort. Postoperative knee radiographs confirmed appropriate prosthesis positioning without evidence of loosening, subsidence, or malalignment (Figure 4). She was encouraged to continue weight-bearing as tolerated and was provided a refill of hydrocodone-acetaminophen (Norco) 7.7-325 mg to take as needed for pain.

At the subsequent two-week follow-up, the patient reported complete resolution of right knee pain and full range of motion. Her only complaint at this visit was worsening pain in the left knee, likely due to compensation and increased weight-bearing on the left leg following surgery. Six weeks postoperatively, the right knee remained stable with only mild tenderness at the incision site. She was eager for definitive treatment of the left knee, which was markedly tender to palpation and with extension. After discussion, she elected to undergo the same procedure on the left knee following her success and satisfaction with the right knee. The same instructions regarding preoperative and postoperative care were provided, and the patient expressed understanding. 

**Figure 5:**
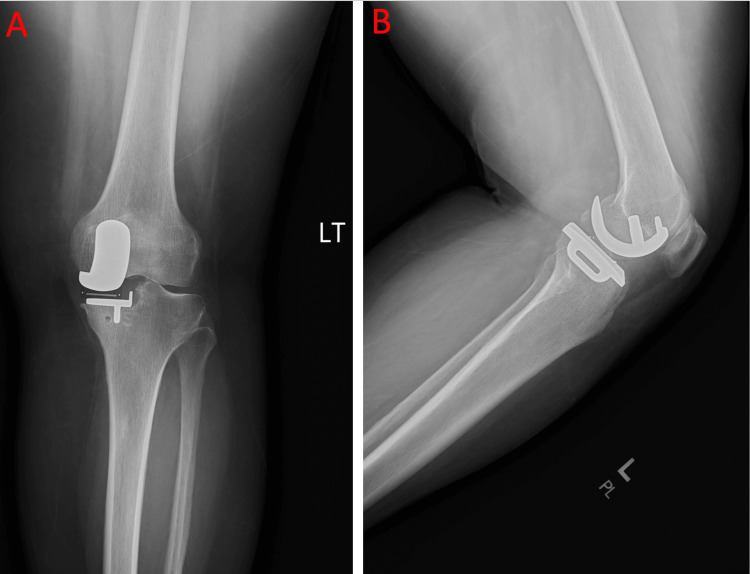
a) Postoperative anteroposterior view of the left knee showing an intact prosthesis without evidence of loosening or migration. b) Postoperative lateral view of the left knee showing an intact prosthesis without evidence of loosening or migration.

At her two-week postoperative follow-up for the left OUKA, the patient reported excellent outcomes, describing only mild medial tightness with ambulation. Physical examination demonstrated no tenderness to palpation, flexion to 115°, and full extension. Postoperative radiographs showed well-positioned hardware without evidence of loosening or migration (Figure 5). A referral for postoperative physical therapy was provided to optimize strength and range of motion during recovery. She was not provided a refill of hydrocodone-acetaminophen, as she felt it was unnecessary and had remaining medication from her prior prescription to use as needed. 

Overall, the patient was completely satisfied with her decision to undergo this procedure. Her pain had improved significantly since the initial visit and continued to progress well. She will continue to be monitored regularly to evaluate her recovery and assess the durability of the hardware in both knees.

## Discussion

The OUKA is an alternative surgical technique aimed at treating isolated medial compartment osteoarthritis and is thought to offer a faster recovery period and a minimally invasive approach [1,3]. First approved in the United States in 2004, many questioned the reliability of the replacement due to high failure rates associated with aseptic loosening, persistent pain, revision surgeries, and conversion to TKAs [3]. Recent improvements have targeted these issues by transitioning toward a cementless design and the use of a fully congruent polyethylene mobile bearing.

The comparison between cementless and cemented approaches for partial knee arthroplasty has been debated for many years. Prior studies from 2013 and 2020, among others, found that uncemented fixation was not inferior to cemented fixation [8,9]. However, a more recent study from 2024 found that the cementless approach showed better 10-year implant survival and postoperative function compared with cemented UKA [6]. Patients who undergo this procedure are more likely to forget their artificial joint in daily life and report greater satisfaction with daily function and activities [10]. For surgeons seeking procedural efficiency, the cementless approach is desirable because it requires a shorter operative time while providing a less invasive approach with improved outcomes [8,11]. The 2024 FDA-approved cementless OUKA also contains a titanium and hydroxyapatite coating, which may further promote bone growth into the implant [12].

In theory, the advantages of cementless OUKA include bone stock preservation, long-term biological fixation to the implant, and avoidance of cementation-related complications. Cementation limits the ability to remodel, potentially leading to higher revision rates after years of repetitive wear and tear [9]. Another benefit is the shortened operative time. Although older studies demonstrated little difference in complication and revision rates between uncemented and cemented UKA, the 2024 study appears to favor the uncemented approach [6,8,9].

Although cementless OUKA has been widely used in the United Kingdom and Europe, recent FDA approval for use in the United States allows greater opportunity for continued research into the evolving debate of cementless versus cemented OUKA. Use in the United States will likely continue to increase in the coming years, and, as this case report demonstrates, early results are promising. In light of new findings and varying conclusions compared with prior studies, continued follow-up and observation of postoperative outcomes remain beneficial.

## Conclusions

This case demonstrates the excellent early outcomes achievable with the newly FDA-approved cementless OUKA. The patient’s rapid recovery, complete resolution of knee discomfort, and high satisfaction make this procedure a potential advantage in the evolution of this technique. The cementless design provides the added benefits of bone stock preservation, avoidance of cement-related complications, and potential for long-term biological fixation through osseointegration, which may translate to improved implant longevity and reduced revision rates compared to traditional cemented techniques. Moreover, the shorter operative time and less invasive approach make it a favorable option for appropriately selected patients. Continued clinical evaluation will be crucial in determining whether cementless OUKA can truly shift the paradigm toward more durable, patient-centered joint preservation strategies.
